# A ubiquitinated E2-based mRNA vaccine confers complete protection against lethal challenge with classical swine fever virus

**DOI:** 10.1128/jvi.00269-26

**Published:** 2026-06-12

**Authors:** Huanjie Zhai, Yuxuan Gao, Nian Wan, Yuxin Qu, Su Li, Lian-Feng Li, Ting Zhao, Qinghe Hou, Yuan Sun, Hongxia Wu, Yongfeng Li, Hua-Ji Qiu

**Affiliations:** 1 State Key Laboratory of Animal Disease Control and Prevention, National High Containment Facilities for Animal Diseases Control and Prevention, Harbin Veterinary Research Institute, Chinese Academy of Agricultural Sciences (CAAS) 687216 , Harbin, People's Republic of China; University of North Carolina at Chapel Hill, Chapel Hill, North Carolina, USA

**Keywords:** classical swine fever virus, E2 protein, mRNA vaccine, antigen modification, ubiquitination

## Abstract

Classical swine fever (CSF), caused by classical swine fever virus (CSFV), continues to threaten the global swine industry. Current E2-based subunit vaccines are limited by their slow adaptation to emerging viral strains and insufficient cell-mediated immunity. To address these challenges, we developed mRNA-lipid nanoparticle (LNP) vaccines encoding modified forms of the CSFV E2 glycoprotein. In this study, we constructed three novel mRNA-LNP vaccines encoding engineered E2 proteins: E2-mRNA-LNP, XCL1-E2-mRNA-LNP containing the X-C motif chemokine ligand 1 (XCL1) to target dendritic cells, and Ub-E2-mRNA-LNP containing ubiquitin (Ub) to enhance antigen presentation through Ub-mediated degradation. These vaccines were evaluated in rabbits and piglets. Following challenge with the live attenuated CSFV C-strain, rabbits immunized with XCL1-E2-mRNA-LNP or Ub-E2-mRNA-LNP exhibited neither fever nor detectable viral RNA in the spleens. Notably, immunization with Ub-E2-mRNA-LNP conferred complete protection against challenge with the highly virulent CSFV Shimen strain in piglets, outperforming the E2-based subunit vaccine, the unmodified E2-mRNA-LNP, and XCL1-E2-mRNA-LNP. Further analysis revealed that Ub-E2-mRNA-LNP elicited more potent antibody and cell-mediated immune responses. Our study demonstrates that Ub-E2-mRNA-LNP is a promising vaccine candidate for CSF prevention.

**IMPORTANCE:**

CSF remains a major threat to the global swine industry. Recently, a moderately virulent subgenotype CSFV 2.1c strain was isolated and reported in China. Given that the mRNA platform enables rapid vaccine development to address emerging pathogens, we constructed three nucleoside-modified mRNA-LNP vaccines expressing engineered E2 glycoproteins of CSFV, including E2-mRNA-LNP, XCL1-E2-mRNA-LNP (targeting dendritic cells), and Ub-E2-mRNA-LNP (enhancing proteasomal degradation). Evaluated in rabbits, XCL1-E2- and Ub-E2-mRNA-LNPs conferred strong protection against CSFV C-strain challenge, with no fever or detectable virus in tissues. Both induced stronger antibody responses than E2-mRNA-LNP. Notably, Ub-E2-mRNA-LNP provided complete protection in piglets, superior to the E2-based subunit vaccine, E2-mRNA-LNP, or XCL1-E2-mRNA-LNP groups and eliciting robust humoral and cellular immunity. These results highlight Ub-E2-mRNA-LNP as a highly promising candidate for an effective CSF vaccine.

## INTRODUCTION

Classical swine fever (CSF), caused by classical swine fever virus (CSFV), is a highly contagious disease that continues to impose substantial economic burdens on global swine production (1, 2). CSFV remains endemic in parts of Asia, Europe, and Africa, causing recurrent outbreaks and trade restrictions (3, 4). The lapinized Chinese strain (C-strain) is considered one of the most effective vaccines for controlling CSF in pigs worldwide (5). Furthermore, the C-strain confers cross-protection against emerging subgenotype 2.1 variants, including CSFV of subgenotypes 1.1, 2.1, 2.2, and 2.3 (6–9). Notably, two emerging strains GD-2024 (subgenotype 2.1c) and HLJZZ2014 (subgenotype 2.1d) have been recently isolated in southern and northeastern China, respectively. Infection with these strains leads to persistent fever, high viremia, and systemic inflammation in affected animals, complicating current CSF control strategies (10–12). Although live-attenuated vaccines (LAVs) effectively induce both neutralizing antibodies (NAbs) and cell-mediated immunity, they do not enable the eradication of CSF when used alone. This is because LAV-induced antibody responses are serologically indistinguishable from those caused by natural infection, making differentiation between infected and vaccinated animals (DIVA) impossible (13).

The E2 glycoprotein, a major envelope protein of CSFV, is the primary target of NAbs and exhibits 90.3% to 95.7% conservation across different strains. Consequently, E2 protein has been extensively utilized in subunit vaccine development, including novel marker E2-based subunit vaccines (14, 15). However, conventional E2-based subunit vaccines and the CSFV C-strain are constrained by critical limitations, including dependency on potent adjuvants to enhance immunogenicity, insufficient induction of robust cellular immunity, and the requirement for multiple immunizations (13). Additionally, E2-based subunit vaccines offer the advantage of DIVA compatibility although their protective efficacy remains lower than that of LAVs.

As professional antigen-presenting cells (APCs), dendritic cells (DCs) internalize antigens and present processed peptides via major histocompatibility complex class I or II (MHC-I or MHC-II) molecules to activate naïve T cells, thereby initiating antigen-specific adaptive immunity (16). Inspired by this mechanism, ubiquitin (Ub)-targeted antigen delivery has been widely employed to develop T-cell-based vaccines against various viral pathogens. The N-terminal fusion of antigens to a G76A-mutated Ub sequence promotes proteasomal degradation, facilitating rapid generation of immunogenic peptides and their efficient entry into the MHC-I antigen presentation pathway, including cross-presentation. This process enhances APCs-mediated antigen presentation (17–20). The binding of X-C motif chemokine ligand 1 (XCL1) to its receptor XCR1 on DCs enhances capture, processing, and presentation of antigenic peptides by MHC-I to CD8^+^ T cells, thus promoting cell-mediated immune responses (21–24).

mRNA vaccines utilize host translational machinery to synthesize pathogen-specific antigens, thereby eliciting robust humoral and cellular immune responses (25). The mRNA platform enables rapid vaccine development and scalable manufacturing. Its effectiveness has been proven through the successful global deployment of COVID-19 mRNA vaccines targeting severe acute respiratory syndrome coronavirus 2 (SARS-CoV-2) (26, 27). mRNA vaccines can stimulate both humoral and cellular immune responses and, through host translational machinery, effectively express complex or unstable antigens (28). Lipid nanoparticles (LNPs) have emerged as a novel delivery platform for mRNA vaccines. They encapsulate and protect mRNA within their hydrophobic core, thereby enabling efficient cellular uptake and intracellular release. LNPs also demonstrate an excellent safety profile, high transfection efficiency, and a strong capacity to elicit potent immune responses, making them a highly promising tool for vaccinology (29, 30). This platform has been successfully utilized to develop mRNA vaccines against various infectious diseases, including COVID-19 (31, 32). Consequently, recent advancements in mRNA technology offer a novel approach to CSF vaccine design.

In this study, the human *α-* and *β-globin*-derived untranslated regions (UTRs) were identified to enhance protein expression and employed in subsequent vaccine design. Three nucleoside-modified mRNA-LNPs expressing the CSFV E2 protein were constructed: one encoded the E2 protein without a transmembrane domain (TMD) (E2-mRNA-LNP), the second encoded the XCL1-E2 chimeric antigen (XCL1-E2-mRNA-LNP), and the third encoded the Ub-E2 chimeric antigen (Ub-E2-mRNA-LNP). We systematically evaluated the immunogenicity and protective efficacy of various mRNA-LNPs and the E2-based subunit vaccine in rabbit and piglet models. The results indicated that the E2 mRNA vaccine modified with an N-terminal ubiquitin sequence (Ub-E2-mRNA-LNP) elicited robust humoral and cell-mediated immune responses, conferring complete protection against the virulent CSFV Shimen (CSFV-SM) strain challenge in piglets, compared with unmodified E2-mRNA-LNP, E2-based subunit vaccine, and XCL1-E2-mRNA-LNP.

## MATERIALS AND METHODS

### Cells and viruses

HEK293T and PK-15 cells were maintained in DMEM (catalog no. D6429, Gibco) supplemented with 5% antibiotics-antimycotics (10,000 IU/mL penicillin, 10,000 μg/mL streptomycin; catalog no. 15240-062, Gibco) and 10% heat-inactivated fetal bovine serum (FBS; catalog no. 10270106, Gibco) at 37°C in a 5% CO_2_ incubator. The CSFV C-strain (GenBank accession no. AY805221), the CSFV-SM (GenBank accession no. AF092448.2), and subgenotype 2.1d CSFV strain (HLJZZ2014) were propagated in PK-15 cells (11).

### Vaccine design

The E2-encoding sequence (GenBank accession no. KC597187.1) was cloned into the pLVX-XCL1 or pLVX-Ub expression plasmid. The construct contained the following elements from the T7 promoter to the 3′end: 5′UTR, Kozak sequence, tissue plasminogen activator (tPA) signal sequence, modified gene (*XCL1* or *Ub*), target gene (*E2*), V5 tag, 3′UTR, and poly(A). mRNAs were synthesized by *in vitro* transcription using T7 RNA polymerase, with complete substitution of uridine in UTRs with N1-methyl-pseudouridine-5′-triphosphate (m1ΨTP). The cleancap reagent AG (catalog no. N-7113-10, TriLink) was used to incorporate a Cap 1 structure at the 5′end, enhancing mRNA stability and translation efficiency. Additionally, the ubiquitin sequence was incorporated with a G76A mutation to prevent its cleavage from the fused E2 protein, ensuring efficient proteasomal targeting; a V5 tag was fused to the C-terminus of the construct.

### Protein structure prediction

The three-dimensional structure of the CSFV E2 monomer was predicted using AlphaFold2 implemented via the ColabFold platform. Multiple sequence alignments (MSAs) were generated by searching the UniRef database with MMseqs2. The prediction parameters were set as follows: 24 recycling iterations, model type = “auto,” random seed fixed at 1, and dropout enabled to enhance conformational sampling. All other parameters were kept at their default values. All structural visualizations were carried out using ChimeraX (33).

### Preparation of lipid nanoparticles

The lipid formulation was composed of an ionizable lipid (SM102), 1,2-dierucoyl-sn-glycero-3-phosphocholine (DSPC), cholesterol, and 1,2-dimyristoyl-rac-glycero-3-methoxypoly (ethylene glycol)-2000 (DMGPEG2000) at a molar ratio of 50:38.5:10:1.5 and was dissolved in ethanol. The lipid-ethanol mixture was rapidly mixed with mRNA dissolved in 10 mM acetic acid-sodium acetate buffer. Next, the lipid phase and mRNA solution were co-flowed at a flow rate ratio (FRR) of 1:3, resulting in a total flow rate of 30 mL/min, corresponding to an N:P ratio of 6:1. The resulting mixture was collected and subjected to ultrafiltration (100 kDa molecular weight cutoff) to remove ethanol and exchange the buffer into 20 mM Tris-HCl (pH 7.5). Subsequently, to adjust the mRNA concentration to 60 µg/mL and the sucrose concentration to 10%, an equal volume of PBS solution containing 20% sucrose was added. Finally, the mRNA-LNP was filtered through a 0.22-µm membrane filter and stored at 4°C until use.

### Physicochemical characterization of LNPs

The LNP size distribution of the samples was characterized by dynamic light scattering (DLS) at 25°C using a Zetasizer Nano Pro instrument (Malvern Instruments, UK). The recorded Z-average size was in the range of 40–180 nm, with a polydispersity index (PDI) below 0.3. The LNP zeta potential was measured using a Zetasizer Nano Pro instrument (Malvern Instruments, UK). Additionally, the encapsulation efficiency of mRNA was determined by measuring both total and free mRNA concentrations using the Quant-it RiboGreen RNA quantification assay (catalog no. R11490, Thermo Fisher Scientific) according to the manufacturer’s protocols using an external standard curve.

### Transfection and Western blotting

HEK293T cells in 24-well plates were transfected with plasmids encoding XCL1-E2 (pLVX-XCL1-E2), Ub-E2 (pLVX-Ub-E2), E2 alone (pLVX-E2), or firefly luciferase (pLVX-Fluc). The cells were then incubated at 37°C in an atmosphere of 5% CO_2_ for 48 h. For the Western blotting procedure, both transfected and non-transfected HEK293T cells were harvested and lysed. Primary antibodies, including anti-V5 tag antibody (rabbit polyclonal, Abcam, 1:1,000 dilution) or anti-GAPDH antibody (rabbit polyclonal, Abcam, 1:5,000 dilution), were incubated at room temperature for 2 h. Subsequently, the horseradish peroxidase-conjugated anti-rabbit antibody (donkey polyclonal, Jackson Immuno Research, diluted at 1:20,000) was incubated at room temperature for 1 h. The signal was developed with the tetramethylbenzidine substrate solution (Beyotime) and imaged using an Azure Imaging Systems (Azure Biosystems).

### Immunization and challenge experiment in rabbits

Nineteen 12-week-old New Zealand white rabbits were randomly assigned to five groups. Based on previously reported doses in rabbits, the rabbits were administered via the intramuscular (i.m.) injection with 30 μg of E2-based subunit vaccine (positive control, *n* = 4), 30 μg of E2-mRNA-LNP (*n* = 4), XCL1-E2-mRNA-LNP (*n* = 4), Ub-E2-mRNA-LNP (*n* = 4), or 1 mL of PBS (negative control, *n* = 3) (34). A booster immunization with the same dose was administered 2 weeks after the prime immunization. Serum samples were collected at 0, 7, 14, and 21 days postvaccination (dpv), and cytokine levels in the sera were quantified by enzyme-linked immunosorbent assay (ELISA). At 21 dpv, the rabbits were challenged intravenously via the marginal ear vein with 10^5.0^ TCID_50_ of the CSFV C-strain. Rectal temperatures were recorded every 6 h from 24 to 72 h postchallenge (hpc) to monitor fever responses. All surviving rabbits at study termination were euthanized. The trained personnel euthanized the rabbits after intravenous injection of pentobarbital sodium (20 mg/kg). The death was confirmed by absent corneal reflex and respiratory arrest. All the rabbits were euthanized at 72 hpc, and spleens were collected to quantify viral genome copies by reverse transcription-quantitative PCR (RT-qPCR).

### Immunization-challenge experiment in piglets

Nineteen healthy specific-pathogen-free (SPF) piglets of 4 weeks old and weighing between 11.5 and 13.5 kg were obtained from the Laboratory Animal Center of the Harbin Veterinary Research Institute (HVRI). The piglets were randomly allocated into five groups and vaccinated via the i.m. route with the following inocula: E2-based subunit vaccine (*n* = 4, 50 μg per piglet, the commercial E2-based subunit vaccine was administered according to the manufacturer’s recommended single dose), PBS (*n* = 3, 2 mL per piglet), and three different E2-based mRNA-LNPs. The dosage of 100 μg per piglet for each mRNA-LNP vaccine was chosen based on doses used in swine studies employing LNP-formulated mRNA vaccines (35–38). Specifically, the experimental groups consisted of XCL1-E2-mRNA-LNP (*n* = 4), Ub-E2-mRNA-LNP (*n* = 4), and E2-mRNA-LNP (*n* = 4). A booster immunization with the same doses was administered 2 weeks after the prime immunization. Serum samples were collected from all piglets at 3, 7, 10, 14, 21, 28, and 42 dpv. The piglets were monitored daily for rectal temperatures and clinical signs, including lethargy, anorexia, depression, vomiting, fever, skin hemorrhages, bloody diarrhea, and joint swelling.

At 28 dpv, all the immunized piglets were challenged with 10^5.0^ TCID_50_/piglet of CSFV-SM (39). EDTA-anticoagulated blood and serum samples were collected at 0, 3, 7, 14, 21, and 28 dpv. Peripheral blood mononuclear cells (PBMCs) were isolated, and the number of IFN-*γ*-secreting cells was determined by enzyme-linked immunospot assay (ELIspot). Inflammatory cytokines were quantified by ELISA. Viral genome copies were measured by RT-qPCR. At 42 dpv, piglets were euthanized under deep anesthesia by intravenous injection of Zoletil (6 mg/kg), followed by bilateral thoracotomy as a secondary measure. After euthanasia, the heart, liver, spleen, lungs, kidneys, and lymph nodes were collected from the challenged piglets.

### ELIspot

The number of IFN-*γ*-secreting T cells was measured using an ELIspot kit (catalog no. 3130-4APW-2, Mabtech), as per manufacturer’s instructions. Briefly, pre-coated plates were treated with RPMI-1640 medium containing 10% FBS (200 μL/well) for 30 min at room temperature. The medium was removed, and 2.5 × 10^5.0^ cells were added per well with 10^5.0^ TCID_50_ of the CSFV. Culture medium was added to the negative control group, and 5 μg of phytohemagglutinin (PHA) mitogen was added to the positive control group. The plates were incubated at 37°C in 5% CO_2_ for 24 h. The cells were discarded, and the plates were washed five times with 200 μL/well PBS. The detection antibody (P2C11-biotin) was diluted to 0.5 μg/mL in PBS containing 0.5% FCS (PBS-0.5% FCS), 100 μL of which was added per well, the mixture was incubated for 2 h at room temperature, and the plates were washed five times with PBS. Streptavidin-HRP (1:1,000 diluted in PBS-0.5% FCS) was added, and the plates were incubated for 1 h at room temperature, followed by five washes with PBS. Next, TMB substrate solution was added and incubated until spots appeared. The reaction was stopped by washing with abundant deionized water. After the plates were dried, the spots were quantified using the ELIspot reader and AID software. IFN-*γ*-secreting T cells per million PBMCs were expressed as spot-forming cells. Negative control as background was subtracted from peptide-stimulated groups, three replicates per group.

### ELISA

All reagents of the commercial CSFV ELISA kit (catalog no. 99-43220, IDEXX) were equilibrated to room temperature. Then, 50 μL of standard substance at various concentrations was added to the standard wells and 50 μL of the test sera was added to the sample wells, while the blank wells were left untreated. Subsequently, 100 μL of detection antibody was added to each well, and all plates were then incubated at 37°C for 2 h. After five washes with wash buffer, substrate A (50 μL/well) and substrate B (50 μL/well) were added to each well and incubated in the dark at 37°C for 15 min. Next, the reaction was halted by adding 50 μL of stop solution per well, and the absorbance was immediately measured at 450 nm (OD_450nm_). A standard curve was plotted using the known concentrations of the standards and their corresponding OD_450nm_ values. The cytokine concentration in each serum sample was then interpolated from this curve. A blocking rate higher than 40%, as defined by the ELISA, was considered positive.

### RT-qPCR

Total RNA was extracted using the RNASimply total RNA kit (catalog no. DP419, Tiangen). RNA was reverse transcribed into cDNA *in vitro* using the Tiangen FastKing gDNA Dispelling RT Supermix kit (catalog no. KR118-03, Tiangen). RT-qPCR was performed to quantify CSFV genome copies in samples, as previously described (40).

### Serum-virus neutralization test

Serum samples were heat-inactivated at 56°C for 30 min prior to analysis. Following inactivation, sera were subjected to two-fold serial dilutions up to a final dilution of 1:12,800. Each diluted serum sample (100 µL) was mixed with an equal volume of CSFV-SM (100 TCID_50_) and incubated at 37°C for 1 h. The virus-serum mixture was then added to PK-15 cell monolayers in 96-well plates. After culturing for 48 h at 37°C with 5% CO_2_, the cells were fixed with ice-cold absolute ethanol for 20 min. Viral infection was determined by sequential incubation with a porcine anti-CSFV primary antibody and a FITC-conjugated rabbit anti-pig IgG secondary antibody (catalog no. F1638-2ML, Sigma-Aldrich), each step followed by washing with PBS. The titers of NAbs were determined as the logarithm of the highest dilution that blocked CSFV infection.

### DIVA

We evaluated the DIVA compatibility of the vaccines using the CSFV E^rns^ antibody ELISA (catalog no. JN60132, JNT). For the test group, diluted serum samples (1:50) were added (100 µL/well) to the E^rns^ antigen-coated wells. Negative controls and positive controls were included by adding 100 µL of SPF pig serum and anti-CSFV serum, respectively, to designated wells. The plate was subsequently incubated at room temperature for 30 min. After incubation, the liquid was discarded, and each well was washed three times with 300 µL of washing buffer. Then, 100 µL of CSFV E^rns^-specific HRP-conjugated antibody was added to each well, and the plate was incubated at room temperature for 30 min. Following another round of aspiration, the wells were washed three times with 300 µL of washing buffer as described above. Next, 100 µL of TMB substrate solution was added to each well, followed by incubation at room temperature for 15 min in the dark. The enzymatic reaction was terminated by adding 50 µL of stop solution to each well. Finally, the absorbance of each well was measured at a wavelength of 450 nm using a microplate reader. The results were calculated based on the predetermined criteria (e.g., sample/negative control ratio or comparison to a standard curve).

### Statistical analysis

Statistical significance was determined using a Student’s *t* test or two-way ANOVA in GraphPad Prism 7. The data were expressed as mean ± standard deviation (SD). Significance is denoted as ns, *P* ≥ 0.05; *, *P* < 0.05; **, *P* < 0.01; ***, *P* < 0.001; ****, *P* < 0.0001.

## RESULTS

### Construction and verification of a mammalian expression vector for the CSFV E2 protein

**Fig 1:**
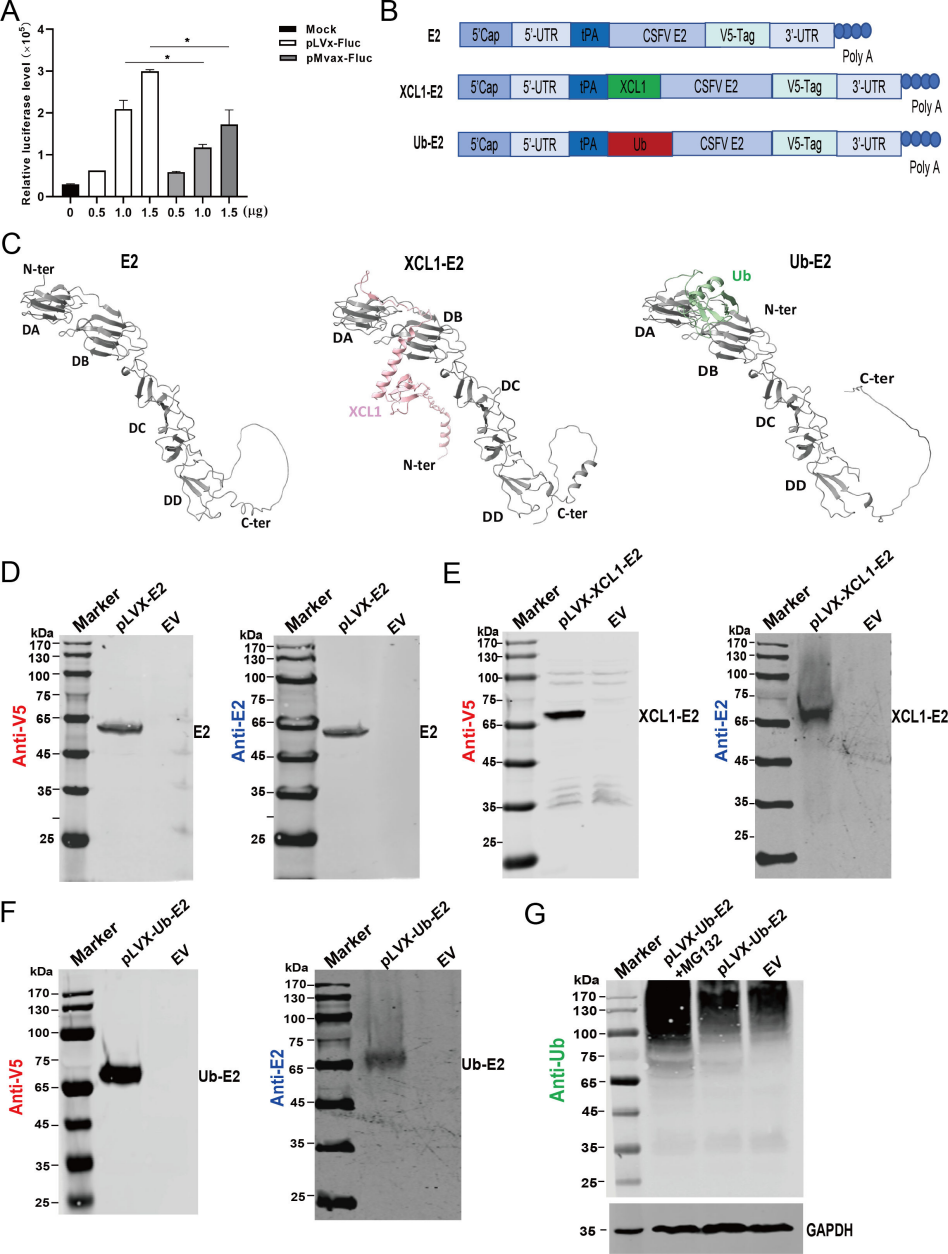
Design and expression of E2-based mRNA vaccines. (**A**) Assessment of UTRs for enhancing mRNA translation efficiency. HEK293T cells were transfected with either the pLVX-Fluc or pMVax-Fluc plasmid, and luciferase activity was measured to evaluate translation efficiency. (**B**) A schematic diagram of mRNA vaccine design. The genes encoding the E2 protein, XCL1-E2 chimeric protein, or Ub-E2 chimeric protein were individually cloned into a template plasmid vector containing 5′cap, 5′UTR, 3′UTR, and 3′poly(A), respectively. (**C**) Comparative analysis of the three-dimensional structural models for different CSFV E2-based antigens. (D−F) The expression of pLVX-E2, pLVX-XCL1-E2, and pLVX-Ub-E2 in HEK293T cells. The expression of E2 (**D**), XCL1-E2 (**E**), and Ub-E2 (**F**) was examined using anti-V5 tag or anti-E2 antibodies. (**G**) Ubiquitination of Ub-E2. Anti-Ub antibody was used to analyze the ubiquitination of Ub-E2 by Western blotting.

The UTRs critically regulate mRNA translation efficiency by modulating stability, ribosome recruitment, and subcellular localization. To systematically identify UTRs configurations that maximize protein expression, we engineered two eukaryotic expression plasmids (pLVX-Fluc and pMVax-Fluc) expressing firefly luciferase (Fluc) under the control of distinct UTRs. pLVX-Fluc incorporated hybrid UTRs derived from human *α*- and *β-globin* genes, whereas pMVax-Fluc contained conventional UTRs and served as a baseline comparator. Luciferase activity assays showed a dose-dependent increase in Fluc expression for both plasmids. Notably, pLVX-Fluc yielded significantly higher Fluc expression than pMVax-Fluc at all doses (Fig. 1A), indicating that UTRs derived from the human *α-* and *β-globin* genes can significantly improve the translation efficiency of mRNA.

It has been reported that the fusion expression of Ub protein with antigen can promote the degradation of antigen by the proteasome, resulting in the rapid production of a large number of the antigen peptides in APCs (14). In addition, XCL1 binding to the XCR1 receptor on DCs recruits these professional APCs to the site of infection, where they can then capture, process, and present antigens to T cells, thereby stimulating cell-mediated immune responses (20). In this study, the antigen was designed based on the E2 protein without the TMDs. Therefore, to improve the ability of mRNA vaccines to stimulate immune responses, the recombinant Ub-E2 protein was constructed in this study. Furthermore, this study is the first to fuse XCL1 with the E2 protein to construct a chimeric antigen XCL1-E2 (Fig. 1B), aiming to improve the efficiency of antigen recognition by DCs. As a control, the template plasmid contained only the E2 protein sequence lacking the TMDs (Fig. 1B). To evaluate the fusion proteins, we confirmed that XCL1 or Ub attached to the N-terminus of E2 does not alter its core conformation (Fig. 1C). The preserved structure allows the XCL1 domain to direct E2 to DCs, and the Ub modification promotes its intracellular processing into antigenic peptides by APCs, thereby leveraging both targeting and processing pathways to consequently potentiate immunity.

The eukaryotic expression plasmids pLVX-E2, pLVX-XCL1-E2, and pLVX-Ub-E2 were transfected into HEK293T cells, respectively. The expression of the E2 protein was analyzed by anti-V5 tag or anti-E2 antibodies. The results showed that E2, XCL1-E2, and Ub-E2 were effectively expressed (Fig. 1D through F). To further verify the ubiquitination of the Ub-E2 protein, HEK293T cells were transfected with the pLVX-Ub-E2 plasmid and treated with or without the proteasome inhibitor MG132 at 8 h posttransfection. Ubiquitinated proteins were observed exclusively in HEK293T cells transfected with pLVX-Ub-E2. MG132 treatment attenuated polyubiquitination, a finding consistent with proteasome inhibition (Fig. 1G). The results suggest that the Ub-E2 protein can be polyubiquitinated through the proteasome pathway.

### Production and characterization of different E2-based mRNA-LNPs

**Fig 2:**
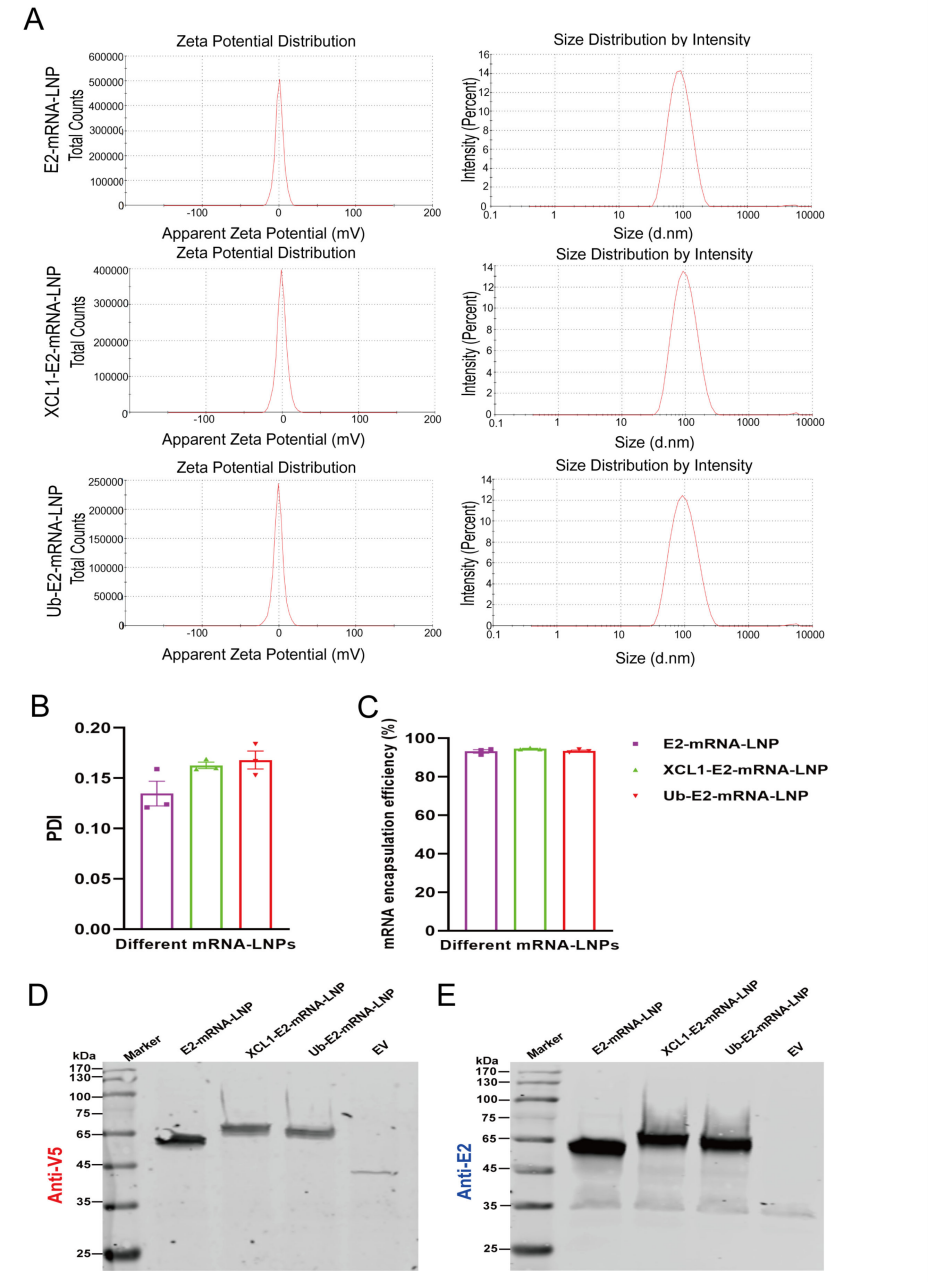
Production and characteristics of different E2-based mRNA-LNPs. (**A–C**) Physicochemical characterization of E2-based mRNA-LNP vaccines. Zeta potential and particle size of different E2-based mRNA-LNPs measured by zeta potential analysis and DLS (**A**), PDI of the mRNA-LNPs (**B**), encapsulation efficiency of the mRNA-LNPs (**C**). (**D and E**) E2 expression of different E2-based mRNA-LNPs. Anti-V5 tag and anti-E2 antibodies were used to detect the E2 expression of different E2-based mRNA-LNPs.

E2-mRNA-LNP, XCL1-E2-mRNA-LNP, and Ub-E2-mRNA-LNP were synthesized by *in vitro* transcription and subsequent lipid nanoparticle encapsulation, using pLVX-E2, pLVX-XCL1-E2, and pLVX-Ub-E2 eukaryotic expression plasmids, respectively. Zeta potential analysis showed that the three mRNA-LNPs exhibited surface charges ranging from −1.90 to 0.223 mV. Additionally, DLS showed that all mRNA-LNP formulations had a particle size between 82.80 and 91.65 nm, with a PDI below 0.2 (Fig. 2A and B). mRNA encapsulation efficiency was consistently above 90% (93.13% for E2-mRNA-LNP, 94.46% for XCL1-E2-mRNA-LNP, and 93.89% for Ub-E2-mRNA-LNP) (Fig. 2C). In summary, all three E2-based mRNA-LNPs exhibited uniform particle size and stable charge.

To further verify the modified E2 expression of E2-mRNA-LNP, XCL1-E2-mRNA-LNP, and Ub-E2-mRNA-LNP, each construct was co-incubated with HEK293T cells for 24 h. Protein samples were collected and subjected to Western blotting analysis. The results showed that high levels of E2, XCL1-E2, and Ub-E2 proteins were expressed in HEK293T cells transfected with the respective mRNA-LNPs (Fig. 2D and E).

### Different E2-based mRNA-LNPs provided complete protection to rabbits against C-strain challenge

**Fig 3:**
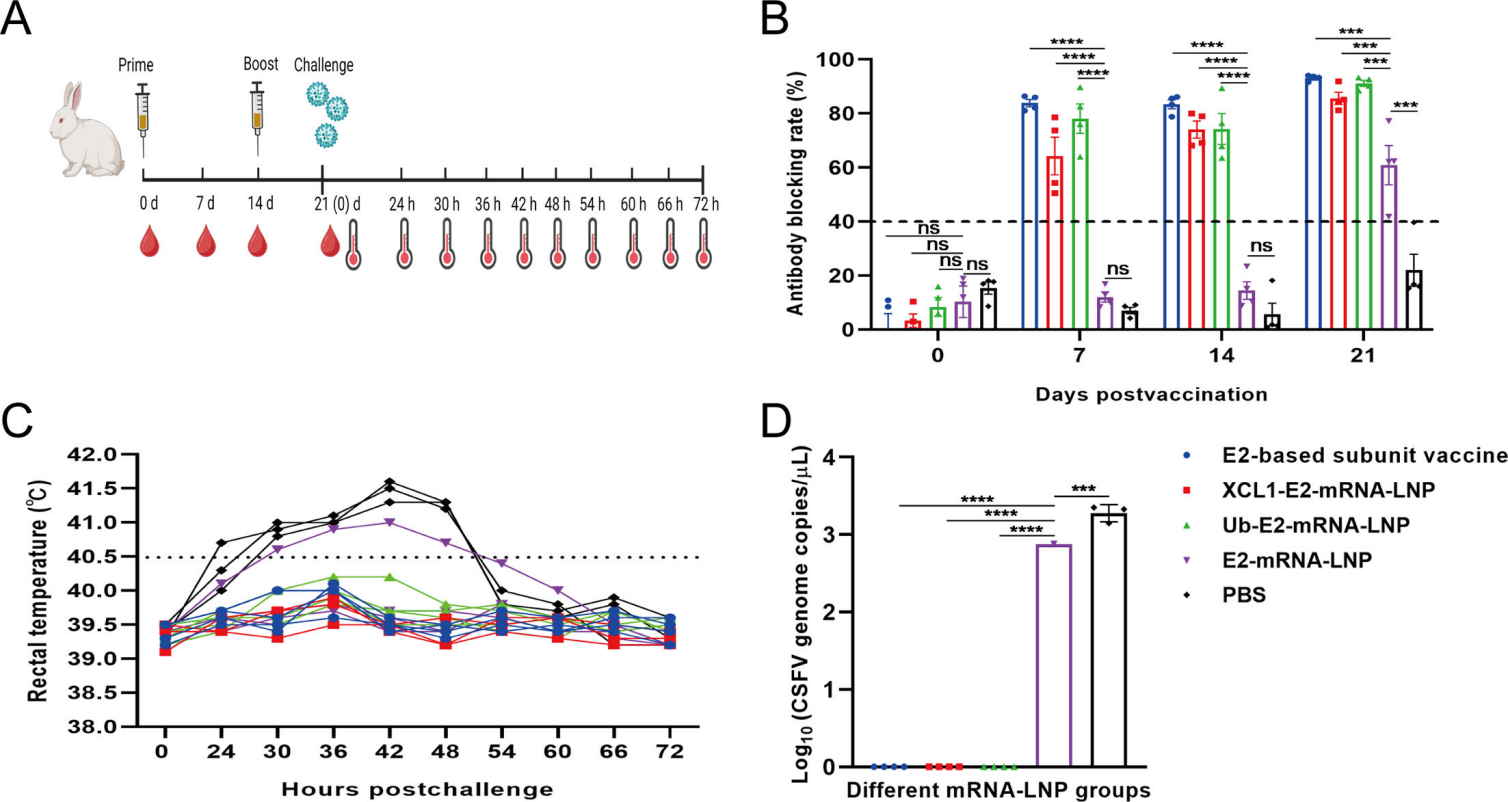
Efficacy evaluation of different E2-based mRNA-LNPs in rabbits. (**A**) Schematic of the rabbit immunization with different E2-based mRNA-LNPs. (**B**) The blocking rates of CSFV-specific antibodies in the sera of the immunized rabbits. An antibody blocking rate higher than 40% was considered positive for anti-CSFV antibodies. (**C**) Rectal temperatures of the immunized rabbits following CSFV C-strain challenge. (**D**) Viral genome copies/mL in the spleens from the challenged rabbits at 72 hpc. The data were expressed as means and SDs. ns, *P* ≥ 0.05; *P < 0.05; **P < 0.01; ***P < 0.001; ****P < 0.0001.

To evaluate the immunogenicity of different E2-based mRNA-LNPs, rabbits were injected by the i.m. route with 30 μg E2-based subunit vaccine, E2-mRNA-LNP, XCL1-E2-mRNA-LNP, Ub-E2-mRNA-LNP, or 1 mL of PBS in the right hind leg of rabbits; the booster immunization was administered following the same procedure at 14 dpv (Fig. 3A). Following the immunization, blocking ELISA demonstrated that serum antibody blocking rates of the rabbits immunized with the E2-based subunit vaccine, XCL1-E2-mRNA-LNP, or Ub-E2-mRNA-LNP were significantly higher than those in the rabbits receiving E2-mRNA-LNP and PBS at each time point. In addition, rabbits immunized with E2-mRNA-LNP seroconverted at 21 dpv, but the blocking rates were significantly lower than those induced by the E2-based subunit vaccine, XCL1-E2-mRNA-LNP, and Ub-E2-mRNA-LNP (Fig. 3B). Collectively, the modified E2-mRNA-LNPs induced significantly higher antibody titers than the unmodified counterpart.

At 21 dpv, all the rabbits were challenged with 100 median infective doses (MID_50_) of the CSFV C-strain. Subsequently, the rectal temperatures of all the rabbits were continuously measured every 6 hpc until 72 hpc. The data showed that rabbits immunized with the E2-based subunit vaccine, XCL1-E2-mRNA-LNP, or Ub-E2-mRNA-LNP did not develop fever. In contrast, one rabbit in the E2-mRNA-LNP immunized group developed fever similar to the fever responses observed in the PBS group (Fig. 3C). Additionally, the viral genome copies were detected in the spleens of the immunized rabbits by RT-qPCR. The results revealed that in rabbits immunized with the E2-based subunit vaccine, XCL1-E2-mRNA-LNP, or Ub-E2-mRNA-LNP, CSFV genome copies were undetectable. Notably, viral RNA was detected in one E2-mRNA-LNP-immunized rabbit and all PBS-control rabbits, confirming that E2-mRNA-LNP provided only partial protection (Fig. 3D). In contrast, rabbits immunized with the E2-based subunit vaccine, XCL1-E2-mRNA-LNP, or Ub-E2-mRNA-LNP were completely protected against CSFV C-strain challenge, as evidenced by the absence of fever and undetectable viremia.

### Piglets immunized with Ub-E2-mRNA-LNP were completely protected from lethal CSFV challenge

**Fig 4:**
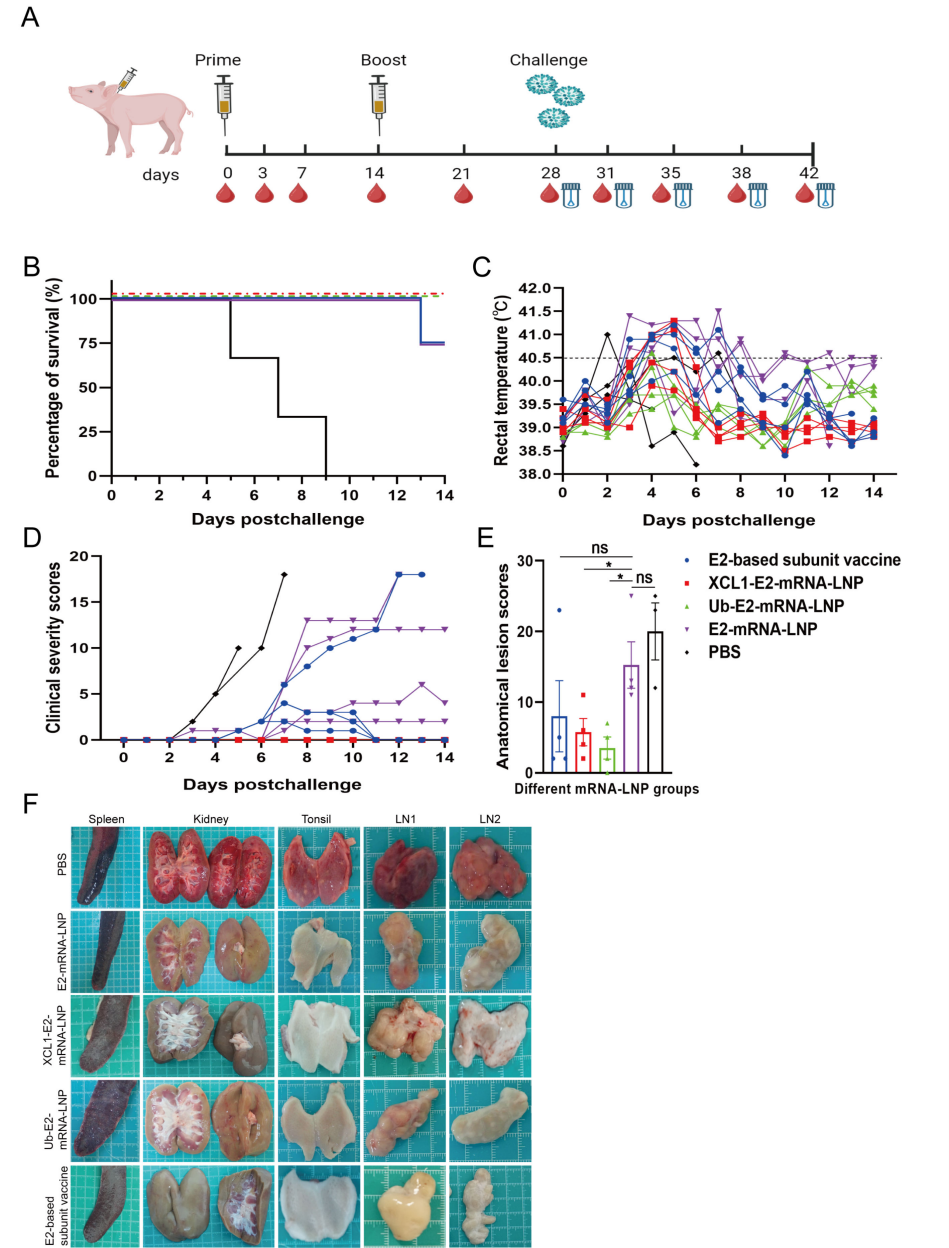
Ub-E2-mRNA-LNP confers complete protection of piglets from lethal CSFV challenge. (**A**) Vaccination scheme with different E2-based mRNA-LNP constructs in piglets. (**B−D**) Evaluation of vaccine efficacy in the immunized pigs challenged with 10^5.0^ TCID_50_ of CSFV-SM. Evaluated parameters included survival rate (**B**), rectal temperature (**C**), and clinical score based on clinical signs typical of CSF (**D**). (**E**) Pathological lesion scores of the immunized piglets following CSFV challenge. (**F**) Representative gross lesions in the immunized piglets following virulent CSFV challenge, including spleen, submandibular lymph nodes, inguinal lymph nodes, kidneys, and tonsils. The data were analyzed using a Student’s *t* test and the two-way ANOVA; bars represent the means ± SD; ns, not significant (*P* ≥ 0.05); *P < 0.05; **P < 0.01; ***P < 0.001; ****P < 0.0001.

To evaluate the efficacy of different E2-based mRNA-LNPs, the piglets were immunized with Ub-E2-mRNA-LNP, XCL1-E2-mRNA-LNP, E2-mRNA-LNP, E2-based subunit vaccine, or PBS. Subsequently, all the pigs were challenged with 10^5.0^ TCID_50_ of CSFV-SM at 28 dpv (Fig. 4A). The survival rate, rectal temperature, and clinical scores were recorded daily for 14 days. Meanwhile, the whole blood, oral swabs, nasal swabs, and anal swabs were collected at 0, 3, 7, 10, and 14 days postchallenge (dpc), and RT-qPCR measured the CSFV genome copies. The results showed that all pigs immunized with Ub-E2-mRNA-LNP and XCL1-E2-mRNA-LNP survived lethal CSFV-SM challenge. One pig in the E2-mRNA-LNP group and one in the E2-based subunit vaccine group died at 13 dpc. Notably, all the pigs injected with PBS succumbed to the infection between 5 and 9 dpc (Fig. 4B). Beyond survival rates, rectal temperature and clinical signs also reflected the protective efficacy of the vaccines. However, pigs immunized with Ub-E2-mRNA-LNP exhibited no elevation in rectal temperature. In contrast, the piglets immunized with XCL1-E2-mRNA-LNP, E2-mRNA-LNP, E2-based subunit vaccine, or PBS developed transient fever (Fig. 4C). The pigs in the E2-mRNA-LNP, E2-based subunit vaccine, and PBS groups began to show typical CSF clinical signs at 3 dpc, such as fever, weakness, anorexia, gait instability, and erythema on the limbs (Fig. 4D).

In addition, the gross pathological examination also confirmed the efficacy of different E2-based mRNA-LNPs. The results showed no significant changes in the tissues and organs of the Ub-E2-mRNA-LNP and XCL1-E2-mRNA-LNP groups. The lymph nodes of the pigs immunized with E2-mRNA-LNP and E2-based subunit vaccine showed slight bleeding. Notably, the PBS group showed severe clinical lesions, including splenic infarction, renal hemorrhage, lymphadenopathy with hemorrhage, and tonsil hemorrhage (Fig. 4E and F).

**Fig 5:**
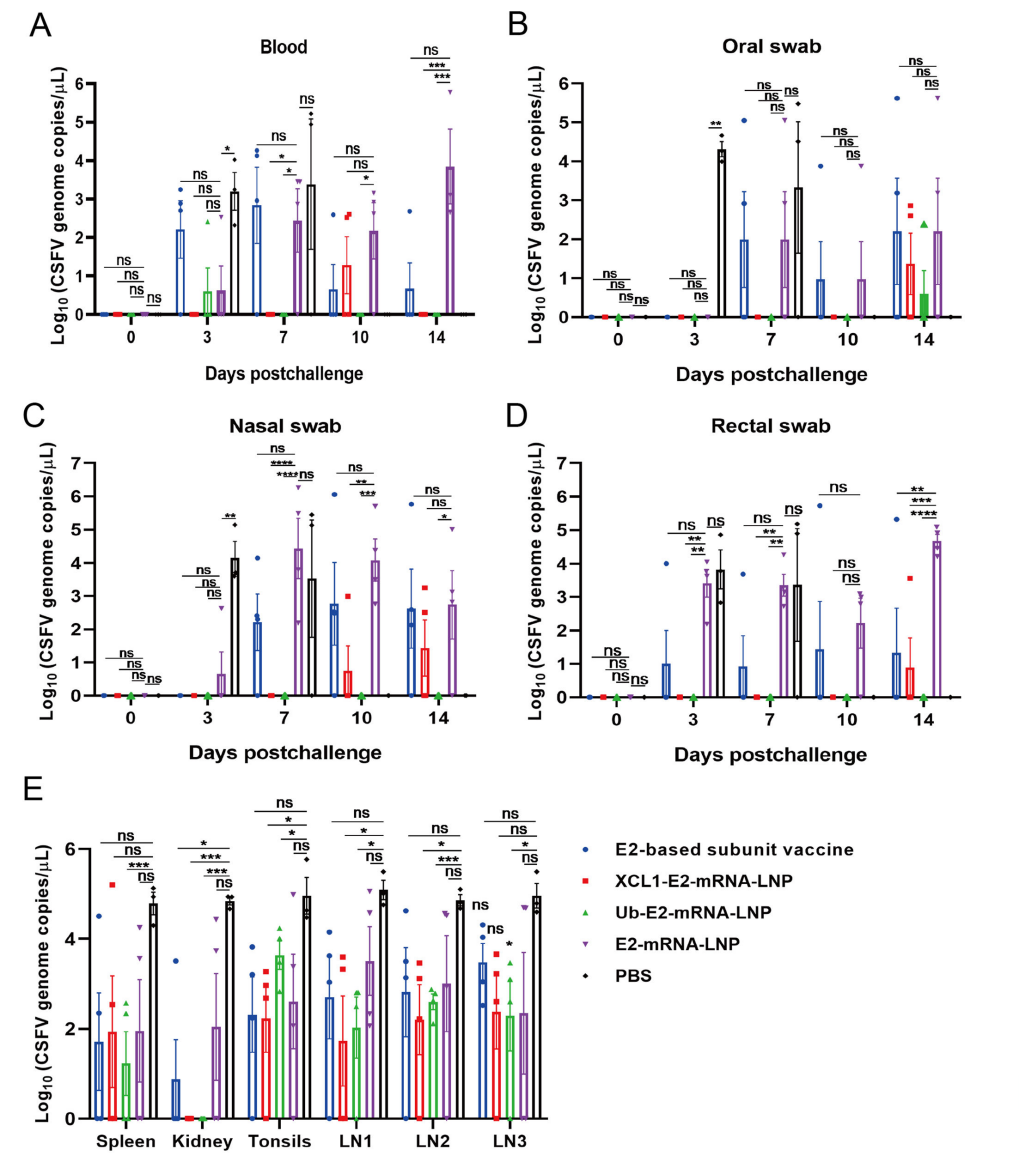
Assessment of viral replication in the immunized piglets following CSFV challenge. Viral replication levels in the blood samples (**A**), oral swabs (**B**), nasal swabs (**C**), rectal swabs (**D**), and tissue samples were systematically collected from the challenged pigs at necropsy (**E**), including the spleen, kidney, tonsils, submandibular lymph node (LN1), mediastinal lymph node (LN2), and gastrohepatic lymph node (LN3). All the tissues were collected from all the infected pigs and subjected to RT-qPCR analysis to quantify the genome copies. The data were analyzed using the two-way ANOVA; bars represent the means ± SD; ns, not significant (*P* ≥ 0.05); *P < 0.05; **P < 0.01; ***P < 0.001; ****P < 0.0001.

Consistent with robust immunity, pigs immunized with Ub-E2-mRNA-LNP or XCL1-E2-mRNA-LNP exhibited no detectable viremia. In contrast, the pigs in the PBS group exhibited high viral loads as early as 3 dpc, with viral titers reaching 10^5.0^ genome copies/mL by 7 dpc, indicating rapid viral replication. The pigs immunized with E2-based subunit vaccine or E2-mRNA-LNP showed lower viremia. At 10 dpc, only one pig in the E2-based subunit vaccine group had viremia (Fig. 5A). Furthermore, no viral RNA was detected in nasal, oral, or rectal swabs from the pigs immunized with Ub-E2-mRNA-LNP. However, in the PBS and E2-mRNA-LNP groups, CSFV RNA was detected in all the swab types. Similarly, pigs immunized with the E2-based subunit vaccine showed limited viral shedding (Fig. 5B through D). These findings demonstrate that following the virulent CSFV challenge, the virus was rapidly neutralized and cleared in the pigs immunized with Ub-E2-mRNA-LNP.

Viral loads in the spleens, tonsils, and lymph nodes of piglets that succumbed to infection were quantified by RT-qPCR. In the pigs immunized with Ub-E2- or XCL1-E2-mRNA-LNPs, viral RNA was detected at low levels. Conversely, the PBS group exhibited viral genome copies exceeding 10^4.0^/mL, indicating substantial viral load (Fig. 5E). These findings confirm that vaccination effectively blocked CSFV replication in major lymphoid organs. These results demonstrate that vaccination with Ub-E2-mRNA-LNP confers complete protection against lethal CSFV challenge in piglets.

### Ub-E2-mRNA-LNP elicited robust immune responses in piglets

**Fig 6:**
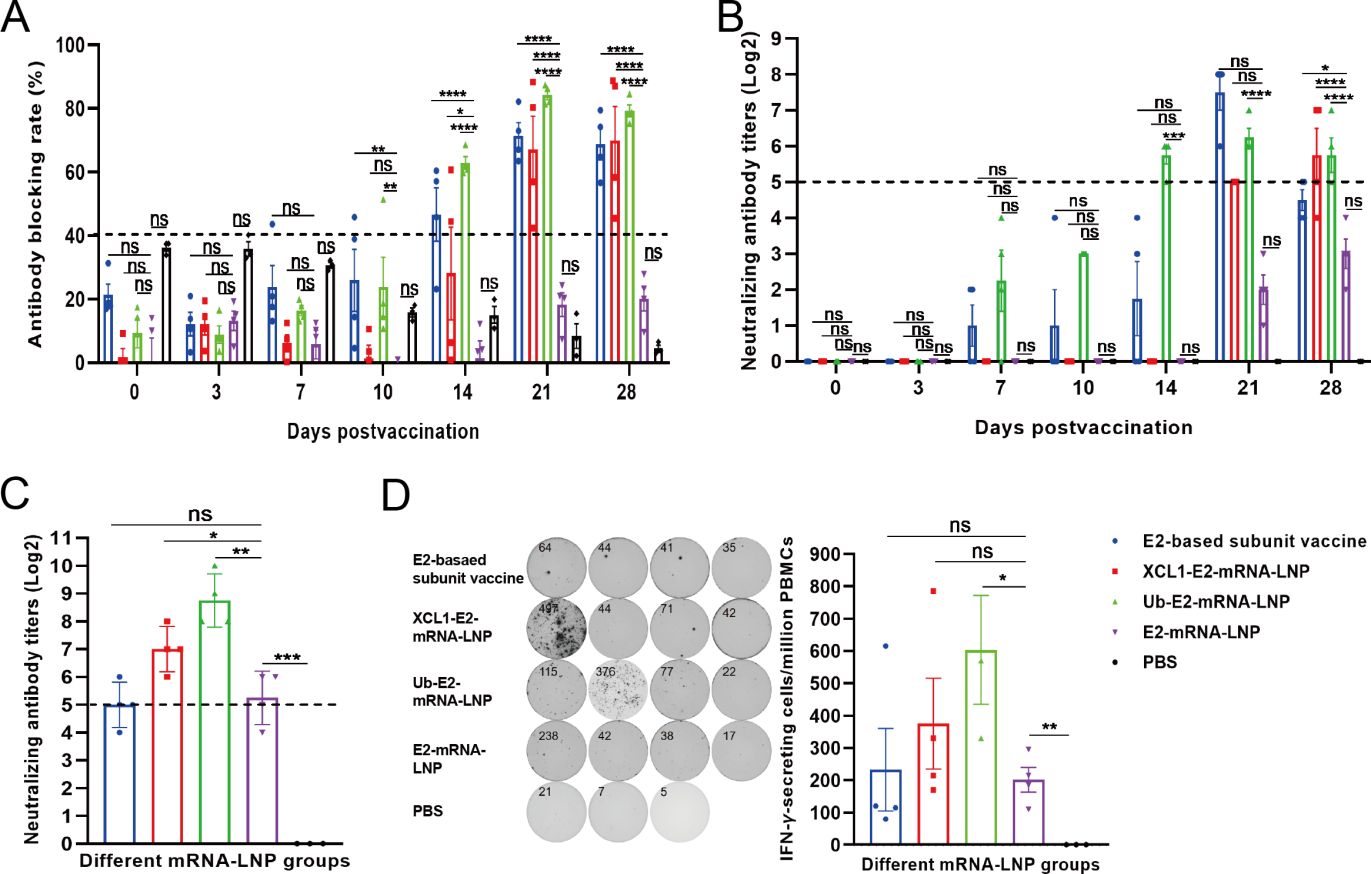
Humoral and cellular immune responses induced by Ub-E2-mRNA-LNP in piglets. (**A**) Antibody responses induced by Ub-E2-mRNA-LNP. The blocking rates of anti-CSFV antibodies in the sera of the immunized piglets were examined by a commercial blocking ELISA kit, with antibody blocking rates higher than 40% defined as positive for CSFV-specific antibodies. (**B**) NAbs in each group at different time points. The titers of NAbs were calculated and expressed as the logarithm of the highest serum dilution that blocked viral infection. A titer higher than 1:32 is considered an indicator of protection (41). (**C**) NAbs against subgenotype 2.1d CSFV in sera collected from different groups. (**D**) Cell-mediated immune responses induced by Ub-E2-mRNA-LNP. CSFV-specific IFN-*γ-*secreting PBMCs from the immunized piglets were quantified by ELIspot. The data were analyzed using the two-way ANOVA; bars represent the means ± SD; ns, not significant (*P* ≥ 0.05); *P < 0.05; **P < 0.01; ***P < 0.001; ****P < 0.0001.

To evaluate the immune responses induced by the different E2-based mRNA-LNPs, the anti-CSFV antibody blocking rates were measured using a commercial blocking ELISA kit. At 10 dpv, one piglet in the Ub-E2-mRNA-LNP group tested positive for CSFV-specific antibodies. Notably, at 14 dpv, the seroconversion rate reached 100% in the Ub-E2-mRNA-LNP group, compared with 50% in the XCL1-E2-mRNA-LNP group and 75% in the E2-based subunit vaccine group. Concurrently, the serum blocking rates were significantly higher in the Ub-E2-mRNA-LNP group than those in the other groups. The XCL1-E2-mRNA-LNP group demonstrated superior late-phase immunogenicity, with higher seroconversion rates than the E2-based subunit vaccine at both 21 and 28 dpv. In contrast, anti-CSFV antibodies were not detectable in the E2-mRNA-LNP group at any time point (Fig. 6A).

There exists a positive correlation between the NAb titer against CSFV and the level of protective immunity. Protective immunity is achieved in pigs once the antibody titer reaches 1:32 (39–41). To assess the neutralizing capacity of vaccine-induced antibodies, sera neutralization titers were evaluated. Sera from the Ub-E2-mRNA-LNP group exhibited neutralizing activity against CSFV-SM as early as 10 dpv, whereas sera from other groups showed no activity at this time point. Furthermore, the peak NAb titers in the Ub-E2-mRNA-LNP group were significantly higher than those in the E2-mRNA-LNP and E2-based subunit vaccine groups (Fig. 6B). Notably, sera from the Ub-E2-mRNA-LNP group effectively neutralized subgenotype 2.1d CSFV strain (Fig. 6C). These results indicate that the Ub-E2-mRNA-LNP vaccine elicits neutralizing activity against CSFV genotypes 1 and 2.

To further assess the CSFV mRNA-LNP-induced CSFV-specific cell-mediated immune responses, PBMCs were isolated from peripheral blood of pigs in the five experimental groups, respectively. The PBMCs were stimulated with the E2 protein and analyzed by ELIspot. The results demonstrated that the Ub-E2-mRNA-LNP group had significantly higher CSFV-specific IFN-*γ*-secreting cells than the control groups. Therefore, the Ub-E2-mRNA-LNP group elicited stronger cellular immune responses than both the E2-based subunit vaccine and conventional E2-mRNA-LNP groups (Fig. 6D).

### Different CSFV-E2 mRNA-LNP constructs demonstrated compatibility with the DIVA strategy

**Fig 7:**
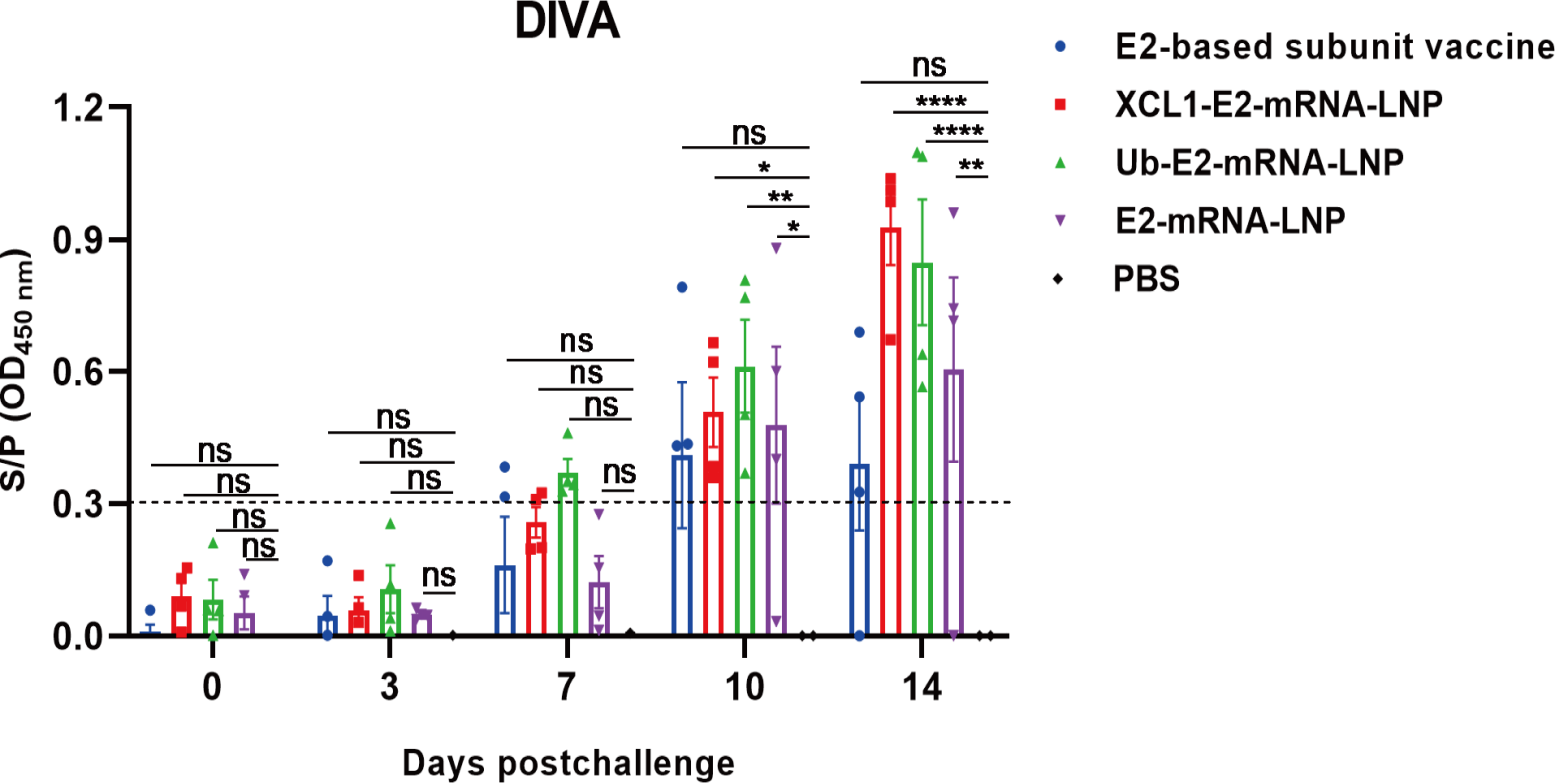
Differentiation of vaccinated from infected pigs based on anti-E^rns^ antibodies. Serum anti-E^rns^ antibodies in different groups following CSFV-SM challenge were examined by an E^rns^-based ELISA. An S/P ratio higher than 30% was considered positive for CSFV E^rns^-specific antibodies. The data were analyzed using the two-way ANOVA; bars represent the means ± SD; ns, not significant (*P* ≥ 0.05); *P < 0.05; **P < 0.01; ***P < 0.001; ****P < 0.0001.

To assess the DIVA compatibility of the CSFV-E2 mRNA-LNP, sera anti-E^rns^ antibody levels were measured by ELISA in different groups postchallenge. At 0 dpc, all the vaccinated pigs were seronegative for anti-E^rns^ antibodies. Following CSFV-SM challenge, both the vaccinated/challenged and PBS control groups seroconverted by 10 dpc, with antibody titers increasing thereafter (Fig. 7). These results demonstrate that vaccination with the CSFV-E2 mRNA-LNP does not induce an anti-E^rns^ antibody response, thereby enabling serological differentiation from infection.

## DISCUSSION

CSF is one of the most devastating diseases affecting the global swine industry (42). Currently, conventional LAVs against CSF remain the primary strategy for its prevention and control. Although most CSF vaccines are effective, safety concerns remain, particularly regarding their use in pregnant sows and piglets (43). Consequently, the lack of serological DIVA capability poses significant challenges to CSF management and eradication efforts (44). Therefore, developing a safer, more effective, and DIVA-compatible vaccine with enhanced immunogenicity is urgently required. The natural TMDs of E2 significantly impair protein expression due to their hydrophobicity. To date, the E2 protein without the TMDs is usually included in CSF subunit vaccines (39). Thus, this study utilized a TMD-deleted E2 variant as the model antigen. Notably, the E2-based mRNA-LNP platform evaluated here offers a promising solution for developing DIVA-compatible CSF vaccines, as antigen production through host translational machinery closely mimics natural viral antigen expression. This approach ensures structural fidelity of the encoded immunogen, including native spatial conformation and posttranslational modifications, which collectively drive the generation of balanced humoral immunity and antigen-specific cellular immune responses (45). Pigs vaccinated with the different CSFV-E2 mRNA vaccines remained seronegative for anti-E^rns^ antibodies. In contrast, following challenge, the vaccinated pigs seroconverted and displayed increasing anti-E^rns^ antibody titers over time postchallenge.

mRNA vaccines offer a promising platform due to their rapid development, scalable manufacturing, and capacity to induce potent immune responses. They show a great potential for preventing and treating various diseases (45, 46). For instance, mRNA vaccines expressing viral antigens, including the SARS-CoV-2 spike protein, influenza virus hemagglutinin protein, respiratory syncytial virus pre-F protein, rabies virus glycoprotein, and porcine epidemic diarrhea virus spike protein have demonstrated complete protection against corresponding viral infections (20, 35, 47–52). As a versatile vaccine strategy, mRNA technology holds immense potential for future immunotherapeutic applications.

The 5′UTR and 3′UTR of mRNA mainly regulate mRNA stability, translation efficiency, nuclear export, and cellular localization, thus directly affecting protein expression (53). The incorporation of the human *α*- and *β*-*globin* 3′UTR sequences has been shown to enhance mRNA stability and translation efficiency markedly. Our results demonstrated that the hybrid human *α-* and *β*-*globin* UTR (pLVX-Fluc) significantly enhanced protein expression compared with conventional UTRs, confirming its utility as a potent regulatory element for optimizing mRNA vaccines. These findings provide a basis for optimizing UTRs in mRNA vaccine design.

The efficiency of antigen uptake by APCs correlated with adaptive immune responses. It has been shown that antigen-specific IFN-*γ* production is essential for preventing CSFV infection (54). However, most of the previously developed CSF subunit vaccines may not induce sufficient cell-mediated immune responses (13). XCL1 mainly functions as a chemoattractant for CD8^+^ DCs expressing the XCR1 receptor. Then, CD8^+^ DCs cross-present antigens to CD8^+^ T cells by MHC-I molecules, promoting the differentiation of CD8^+^ T lymphocytes into cytotoxic effector T cells (55, 56). In this study, the anti-E2 antibody blocking rates in the rabbits immunized with the E2-based subunit vaccine, XCL1-E2-mRNA-LNP, and Ub-E2-mRNA-LNP were significantly higher than those in groups receiving equivalent doses of E2-mRNA-LNP or PBS. Notably, the XCL1-E2-mRNA-LNP formulation elicited significantly enhanced cellular immunity. For the XCL1-E2 fusion construct, the XCL1 moiety directs the E2 antigen to the XCR1 on DCs, thereby facilitating enhanced antigen uptake by APCs. Future studies will focus on optimizing the LNP delivery system to further enhance the vaccine’s stability and immunogenicity and evaluating its cross-protection against diverse CSFV genotypes.

mRNA vaccines harbor genes that synthesize foreign antigens to be presented by MHC molecules in cells. Ubiquitination serves as a canonical signal for proteasomal degradation (57). In mRNA design, ubiquitinated antigenic peptides undergo rapid proteasomal degradation to generate epitopes, which are subsequently presented by APCs via the MHC molecules (13). It has been found that mRNA expressing ubiquitin and tandem epitope-rich peptide (HLA-EP) sequences induces enhanced specific immune responses against SARS-CoV-2 variants (20). Additionally, commercial C-strain-based CSF vaccines of different origins showed molecular variations and antigenic differences and could provide clinical protection against the emerging subgenotype 2.1d CSFV strain that is currently circulating in China (11, 12). Notably, the amino acid identity of the E2 protein between the subgenotype 2.1c and the CSFV-SM strains is 90.3%, while it reaches 95.7% between the subgenotype 2.1c and subgenotype 2.1d CSFV strains (10, 11). In this study, sera from all immunized groups effectively neutralized the subgenotype 2.1d CSFV strain. These findings indicate that the anti-E2 antibodies induced by CSFV-SM vaccination confer robust cross-protective immunity against heterologous strains, including subgenotypes from genotypes 1 and 2. Given the advantage of rapid development, mRNA vaccines represent a promising strategy to combat emergent infectious diseases. Therefore, this study was initiated to develop an mRNA vaccine against CSF (10).

E2-specific antibodies were induced at 35 dpv using a circular mRNA vaccine encoding CD154, E2, and mi3 self-assembled nanoparticles (36). In contrast, immunization with mRNA-LNPs expressing the E2 ectodomain fused with the PEDV S2 protein TMD (E2-tm mRNA-LNP) elicited antibody responses at 14 dpv (37). In order to enhance the capture and processing of antigens by APCs and improve the ability of antigens to induce cell-mediated immune responses, Ub was fused with antigen to form the recombinant antigen Ub-E2. The G76A-mutated ubiquitin fused to E2 is predicted to promote rapid antigen processing into peptides within APCs. These peptides are then efficiently loaded onto MHC class I molecules, thereby potentiating the CD8^+^ CTL response. This mechanism aligns with our observed enhanced IFN-*γ* ELIspot responses in the Ub-E2 group compared with other groups. Furthermore, for the Ub-E2 fusion protein, the ubiquitinated moiety promotes proteasomal degradation of the E2 protein, leading to enhanced antigen processing and MHC presentation. It is worth noting that Ub-E2-mRNA-LNP induced antibody responses at 7 dpv, indicating an even earlier onset of humoral immunity in vaccinated pigs. While XCL1-fusion aims to enhance antigen uptake by specific DCs subsets, the ubiquitin fusion directly amplifies the intracellular antigen processing pathway, leading to a more robust and perhaps qualitatively distinct cellular immune profile that may be critical for achieving protective immunity against CSFV. Collectively, this targeted engagement likely enhances antigen uptake, processing, and presentation by APCs, thereby enhancing cell-mediated immune responses, ultimately boosting vaccine-induced immune protection.

Compared with LAVs, conventional E2-based subunit vaccines typically fail to induce robust immunity, which refers to immunity that prevents pathogen replication and shedding, eliminating the risk of transmission. However, our study demonstrates that this limitation can be overcome by the ubiquitin-modified E2 mRNA-LNP vaccine developed herein. The piglets immunized with E2-mRNA-LNP or the E2-based subunit vaccine exhibited only partial protection against CSFV-SM lethal challenge. Notably, Ub-E2-mRNA-LNP provided complete protection against the same challenge. The virus was rapidly cleared after challenge in the Ub-E2-mRNA-LNP group. Specifically, Ub-E2-mRNA-LNP induced robust early humoral immunity, a result corroborated by mechanistic studies indicating enhanced antigen processing and presentation. Conventional E2-based subunit vaccines require more time to induce NAbs and cannot induce high levels of cellular immunity (15). Crucially, cellular immunity also plays a critical role in protection against CSFV infection. Particularly, the Ub-E2-mRNA-LNP-immunized piglets developed stronger cell-mediated immune responses than the XCL1-E2-mRNA-LNP group, E2-based subunit vaccine group, or PBS group. This is most likely due to the ubiquitin-facilitated proteasomal degradation of the E2 antigen, which enhances the loading of antigenic peptides onto MHC class I molecules, and facilitates cross-presentation by APCs. Proteasome-targeting peptides enhance antigen processing and presentation, potentiating cellular immune responses induced by nucleic acid-based vaccines. Indeed, this strategy has been successfully implemented in multiple vaccine platforms targeting intracellular pathogens (16–18). These findings demonstrate that antigen modification strategies can significantly enhance the immunogenicity and protective efficacy of vaccines.

Although Ub-E2-mRNA-LNP elicited rapid immune responses, its efficacy was evaluated at only a single time point (28 dpv) postchallenge. The early immune response kinetics suggest that protection may be established earlier than 28 dpv. Future studies should, therefore, assess protection at earlier time points postvaccination (e.g., 7 or 14 dpv) to confirm this potential for rapid immunity. Additionally, the present study only evaluated challenge at 42 dpv and terminated monitoring at 14 dpc. Extended observation periods should be considered in future work to enable a more comprehensive assessment of the immune protection conferred by different CSFV-E2 mRNA-LNP formulations. Although mRNA vaccines are expensive compared with conventional vaccines, they are able to induce rapid, robust, and durable immune responses. Our data demonstrate that Ub-E2-mRNA-LNP not only elicits a strong early immune response and complete protection but also significantly reduces viral shedding, likely limiting onward transmission. This combination of rapid immunogenicity and efficacy in blocking spread is critical for emergency control and could translate into net economic benefits at the herd level, mitigating the impact of initial vaccine cost. In summary, despite its higher upfront cost, Ub-E2-mRNA-LNP represents a promising candidate due to its rapid development potential against emerging strains and its ability to induce swift, protective immunity. Further optimization of production processes to achieve economies of scale will be essential to enhance its cost-competitiveness for large-scale livestock use.

In conclusion, we developed a ubiquitin-modified E2-mRNA-LNP vaccine that confers complete protection against lethal CSFV challenge in pigs. This strategy offers a promising DIVA-compatible platform for next-generation CSF vaccines.

## Data Availability

All data are included in the paper. Additional data are available from the corresponding authors on reasonable request.
